# Developmental oxidative stress leads to T-type Ca^2+^ channel hypofunction in thalamic reticular nucleus of mouse models pertinent to schizophrenia

**DOI:** 10.1038/s41380-021-01425-2

**Published:** 2022-01-25

**Authors:** Corinne El Khoueiry, Jan-Harry Cabungcal, Zita Rovó, Margot Fournier, Kim Q. Do, Pascal Steullet

**Affiliations:** Center for Psychiatric Neuroscience, Department of Psychiatry, Lausanne University Hospital, Site de Cery, CH-1008 Prilly-Lausanne, Switzerland

**Keywords:** Neuroscience, Physiology, Schizophrenia

## Abstract

Impairment of parvalbumin interneurons induced by oxidative stress (OxS) is a “hub” on which converge several genetic and environmental risk factors associated with schizophrenia. In patients, this could be a mechanism leading to anomalies of the thalamic reticular nucleus (TRN) whose major neuronal population expresses parvalbumin. The TRN shapes the information flow within thalamo-cortical circuits. The low-threshold voltage-gated T-type Ca^2+^ (T-Ca^2+^) channels (CaV3.2, CaV3.3) contribute to the excitability and rhythmic bursting of TRN neurons which mediates cortical sleep spindles, known to be affected in schizophrenia. Here, we investigated the impact of OxS during postnatal development and adulthood on firing properties and T-Ca^2+^ channels of TRN neurons. In *Gclm* knock-out (KO) mice, which display GSH deficit and OxS in TRN, we found a reduction of T-Ca^2+^ current density in adulthood, but not at peripuberty. In KO adults, the decreased T-Ca^2+^ currents were accompanied with a decrease of CaV3.3 expression, and a shift towards more hyperpolarized membrane potentials for burst firing leading to less prominent bursting profile. In young KO mice, an early-life oxidative challenge precipitated the hypofunction of T-Ca^2+^ channels. This was prevented by a treatment with N-acetylcysteine. The concomitant presence of OxS and hypofunction of T-Ca^2+^ channels were also observed in TRN of a neurodevelopmental model relevant to psychosis (MAM mice). Collectively, these data indicate that OxS-mediated T-Ca^2+^ hypofunction in TRN begins early in life. This also points to T-Ca^2+^ channels as one target of antioxidant-based treatments aiming to mitigate abnormal thalamo-cortical communication and pathogenesis of schizophrenia.

## Introduction

The thalamic reticular nucleus (TRN) is composed of GABAergic neurons that receive synaptic inputs from cortex, thalamus, and subcortical regions and exert inhibition onto thalamic neurons projecting to the cortex or subcortical areas [1–5]. Therefore, it occupies a key position to modulate thalamo-cortical information flow [6]. The TRN is involved in regulation of sleep-arousal states [7, 8], sleep spindles [9] and sleep-mediated memory consolidation [10], sensory filtering and discrimination [11, 12], sensory gating [13], reversal learning requiring the switch from one sensory modality to another [14], modulation of fear extinction [15], flight behavior [16], and search strategies during spatial navigation [17].

Consequently, aberrant thalamo-cortical communication, sleep disturbances, altered sensory perception, deficits of cognitive and emotional processing in schizophrenia (SZ) patients could potentially result from a TRN dysfunction. The TRN of SZ patients displays abnormal perineuronal net (PNN) and reduced density of parvalbumin (PV)-immunoreactive neurons [18], which represent the larger neuronal population of the TRN and regulate sleep spindles [19] affected in SZ [20, 21]. Furthermore, individuals with early psychotic episodes [22] and SZ patients [23] have been reported with low GSH levels in thalamus and anterior cingulate cortex. Similarly, an animal model of redox dysregulation pertinent to SZ, *Gclm*-KO mouse, shows PNN and PV cell anomalies in TRN and brain GSH level deficit [24] due to the knockout of the modulatory subunit (GCLM) of the key GSH synthesizing enzyme [25]. TRN neurons of *Gclm*-KO mice are particularly prone to oxidative stress (OxS) [24] that also mediates decreased PV expression in TRN of NMDAR hypofunction and neurodevelopmental animal models relevant to SZ [26, 27]. Moreover, TRN neurons of *Gclm*-KO mice are less inclined to generate bursts of action potentials as compared to WT mice [24]. However, the underlying molecular mechanism causing altered TRN neuron excitability in *Gclm*-KO mice and its consequences on thalamo-cortical connectivity [28] remains unclear.

One remarkable feature of TRN neurons is their capacity to display either a tonic or phasic activity according to the activation/inactivation characteristics of low voltage-activated T-type Ca^2+^ (T-Ca^2+^) channels [29]. Furthermore, the coordinated activation of T-Ca^2+^ channels and small-conductance Ca^2+^-activated K^+^ (SK) channels causes repeated burst firing essential for sleep spindle generation during NREM sleep [30–32]. TRN neurons bear two subtypes of T-Ca^2+^ channels, namely CaV3.2 and CaV3.3. Genome-wide association studies have revealed strong link between the gene encoding CaV3.3, CACNA1I, and schizophrenia [33]. While CaV3.3 contribute largely to the burst firing properties and rhythmic bursting of TRN neurons, CaV3.2 boost excitability and intrathalamic transmission mediated through burst discharges [32]. Therefore, we hypothesized that OxS-mediated T-Ca^2+^ channel hypofunction is at the origin of the altered firing property of TRN neurons in *Gclm*-KO mice and constitutes one pathological mechanism relevant to SZ.

Using whole-cell patch clamp techniques, we found reduced T-Ca^2+^ currents in TRN neurons of young adult, but not peripubertal *Gclm*-KO mice. Oxidative insult during early-life however precipitated this hypofunction in peripubertal KO mice which was prevented by N-acetyl-cysteine. We also observed a similar association between OxS and decreased T-Ca^2+^ currents in TRN neurons of mice with an aberrant neurodevelopment induced by a prenatal injection of methylazoxymethanol acetate (MAM). These suggest that OxS-mediated T-Ca^2+^ channel hypofunction during early-life represents a common, convergent mechanism contributing to thalamo-cortical anomalies, sleep disturbances [28], and possibly risk for psychosis.

## Methods

### Animal models

Unless indicated, the effect of developmental oxidative stress was assessed in males and females from different mouse models. Experiments were approved by the Local Veterinary Office.

#### Gclm-KO mice as described in [25]

See Supplemental information for details.

#### GBR-treated Gclm-KO mice and GBR + BSO-treated WT mice

From postnatal days (PND)10 to PND20, the dopamine reuptake inhibitor GBR-12909 (GBR, Bio Trend, Germany) was injected daily (5 mg/kg, i.p) in *Gclm*-KO mice and every other day in WT mice who received a daily injection (3.8 mmol/kg, s.c.) of the specific inhibitor of GSH synthesis, buthionine sulfoximine (BSO, Sigma-Aldrich, Switzerland).

#### MAM mice

Female C57BL/6JRj mice were injected intraperitoneally on gestational day 16 with either 25 mg/kg methylazoxymethanol acetate (MAM, MRIGlobal Chemical Carcinogen, USA) or 0.9% NaCl. The offspring of MAM- or NaCl-treated females were used for experiments.

#### N-Acetylcysteine (NAC) treatment

NAC (Fluimucil, Zambon, Switzerland) was given to GBR-treated *Gclm*-KO mice from PND2 to sacrifice (Supplemental information).

### Electrophysiology experiments

We prepared brain slices from peripubertal (PND20-30) and young adult (PND60-75) mice and performed patch-clamp whole-cell recordings of TRN neurons in both current clamp (CC) and voltage clamp (VC) modes as described in Supplemental information. We determined the basic membrane properties, the firing pattern (bursting versus tonic) at resting membrane potential, and the threshold for the initial membrane potential required to induce single or repetitive bursting upon depolarization using current-injected protocols described in Supplemental information. A burst was defined as a high-frequency phasic activity (interspike frequency > 100 Hz) followed by an afterhyperpolarization period. We isolated T-Ca^2+^ currents and SK currents using VC protocols described in [29] and Supplemental information.

### Immunohistochemistry

We prepared brain sections and performed parvalbumin (PV)/Wisteria floribunda agglutinin (WFA)/8-hydroxy-2′-deoxyguanosine (8-oxo-dG) triple immunofluorescence on males as described in [18] and Supplemental information. Primary antibodies were mouse monoclonal anti-8-oxo-dG (AMS Biotechnology, Switzerland), rabbit polyclonal anti-PV (Swant, Switzerland), biotin-conjugated lectin WFA (Sigma-Aldrich, Switzerland), while secondary antibodies were goat anti-mouse AF488 (Life Technologies, USA), goat anti-rabbit IgG CY3 (Chemicon International, USA) and streptavidin 405 conjugate (Millipore Corporation, USA).

We carried out CaV3.2/PV and CaV3.3/PV immunohistology as indicated in Supplemental information using the following primary [rabbit anti-CaV3.2 (Santa Cruz Biotechnology, USA); rabbit anti-CaV3.3 (Alomone labs, Israel), sheep anti-PV (R&D systems)] and secondary [goat anti-rabbit AF488 and donkey anti-sheep AF594 (Abcam, UK)] antibodies. Confocal image acquisition, and quantification are described in Supplemental information.

### Statistics

See Supplemental information.

## Results

### Altered bursting profile of TRN neurons in adult *Gclm*-KO mice

**Fig. 1:**
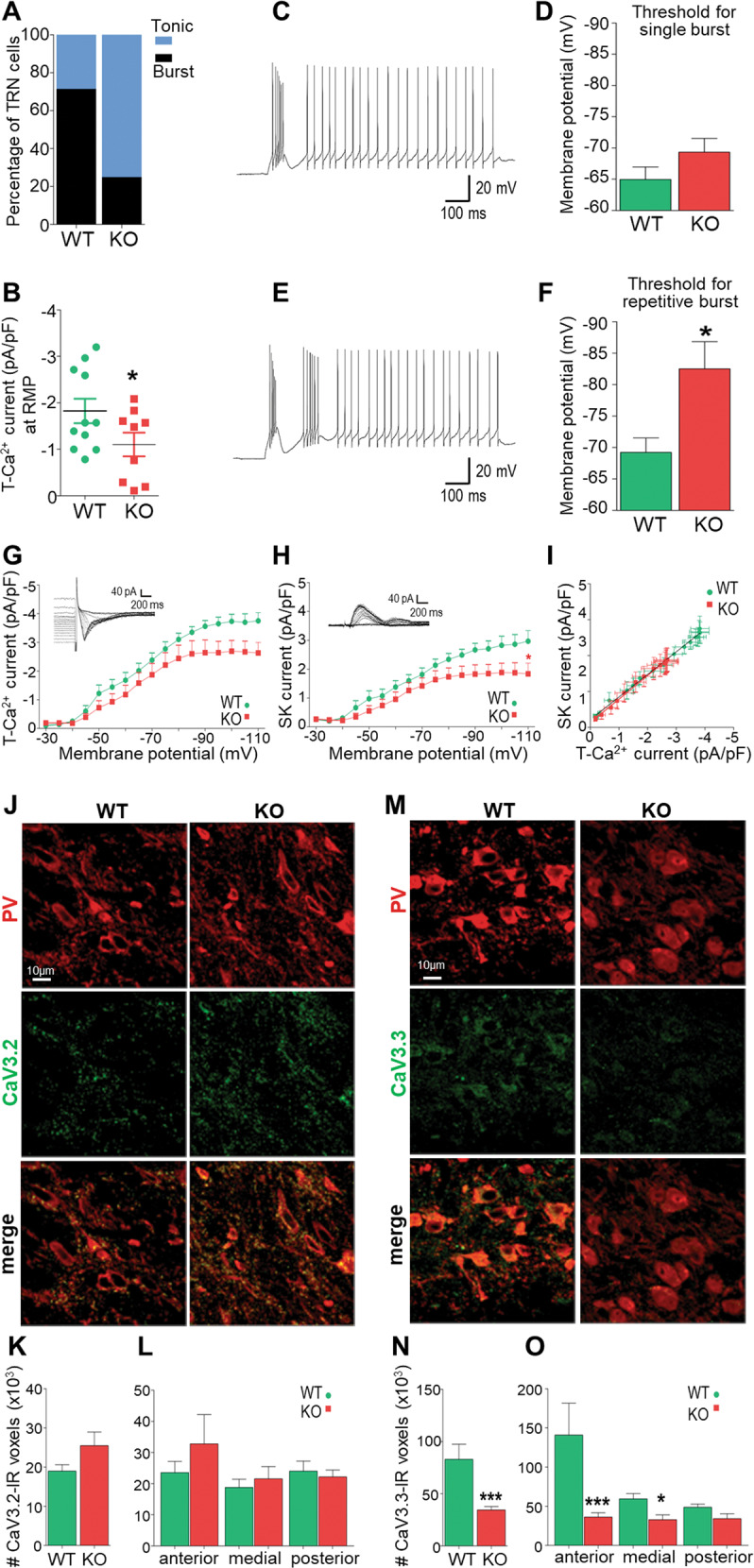
Hypofunction of T-Ca^2+^ channels and decreased CaV3.3 expression in TRN neurons of adult *Gclm*-KO mice, resulting in alteration of their bursting profile. **A** Smaller proportion of TRN neurons generating burst firing at resting membrane potential (RMP) in KO as compared to WT mice (WT *n* = 13; KO *n* = 12; Fisher exact test, *p* = 0.02). **B** Weaker density of T-Ca^2+^ currents activated at RMP in TRN neurons of KO (*n* = 11) as compared to WT (*n* = 10) mice (*p* = 0.034, one-tailed *t*-test). **C** Representative recording of single bursting in a TRN neuron. **D** Threshold for the initial membrane potential required to induce single bursting upon depolarization. **E** Representative recording of repetitive bursting in a TRN neuron. **F** Threshold for the initial membrane potential required to induce repetitive bursting upon depolarization. Note that KO (*n* = 6) TRN neurons require a more hyperpolarized membrane potential, particularly for exhibiting repetitive bursts, as compared to WT mice (*n* = 8). **G**, **H** Top: Representative recordings of T-Ca^2+^ and SK currents induced by a short constant depolarization step (from −110 to −40 mV), with their amplitudes increasing with greater hyperpolarizing initial membrane potential (going from −30 mV to −110 mV). Bottom: Density of T-Ca^2+^ (**G**) and SK currents (**H**) activated from each of the initial membrane potentials. Compared to WT mice, TRN neurons in KO display overall smaller T-Ca^2+^ (*F* = 31.92 DF*n* = 1 DFd = 289; *p* < 0.0001; WT *n* = 9; KO *n* = 10) and SK current densities (*F* = 36.34 DF*n* = 1 DFd = 289; *p* < 0.0001; WT *n* = 9; KO *n* = 10). **I** Correlation between T-Ca^2+^ and SK current densities in TRN neurons of both genotypes (Spearman test, *r* = −0.99 for WT; *r* = −1 for KO; *p* < 0.0001 for both). **J** Micrographs showing immunofluorescent labeling for parvalbumin (PV, red) and CaV3.2 (green) in the TRN of adult WT and KO mice. **K** No significant difference of CaV3.2 labeled profiles (number of CaV3.2-IR voxels) in TRN of WT and KO mice (*n* = 5 for both genotypes). **L** Regional CaV3.2 quantification within the anterior, medial, and posterior sections of the TRN. **M** Micrographs showing immunofluorescent labeling for PV (red) and CaV3.3 (green) in the TRN of adult WT and KO mice. **N** Reduced CaV3.3 labeled profiles (number of CaV3.3-IR voxels) in TRN of KO as compared to WT mice (*n* = 4 for both genotypes). **O** Reduced expression of CaV3.3 mostly in the anterior and medial sections of the TRN in KO as compared to WT mice. Unpaired t-tests or two-way ANOVAs with Bonferroni post-tests; *p < 0.05 **p < 0.01 ***p < 0.001. Data are presented as means ± s.e.m.

We first characterized the bursting profile of TRN neurons in young adult (PND60-75) mice using whole-cell current-clamp techniques. We found a lower proportion of neurons displaying bursting behavior at resting membrane potential (RMP) in KO (25.0%, 3/12) compared to WT mice (71.4%, 10/13, Fisher exact test, *p* = 0.02) (Fig. 1A), while neurons from both genotypes showed similar resting membrane properties (RMP: −59.5 ± 2.5 vs −63.4 ± 2.4 mV; series resistance: 364.4 ± 48.4 vs 329.4 ± 30.7 MΩ; membrane capacitance: 46.2 ± 3.8 vs 49.7 ± 4.1 pF in KO and WT, respectively). Concomitantly with the less prominent bursting profile of TRN neurons of KO mice at RMP, the T-Ca^2+^ current density at RMP was significantly weaker in KO mice (Fig. 1B). Since the burst firing behavior increases with more negative membrane potentials due to the inactivation properties of T-Ca^2+^ channels, we then determined the membrane potential at which TRN neurons switch from tonic to burst firing. We applied a series of incremental hyperpolarizing currents followed by a depolarizing step, and found that the switch from tonic to bursting occurred at a membrane potential of −69.3 ± 2.2 mV in KO as compared to −64.9 ± 1.9 mV in WT mice (Fig. 1C, D). The membrane potential required for repetitive bursting was significantly more negative in KO (−82.5 ± 4.3 mV) compared to WT mice (−69.2 ± 2.3 mV; *p* = 0.02, Fig. 1E, F). Collectively, this indicates that TRN neurons in adult KO mice displayed a bias for tonic firing at conditions close to RMP.

### Decreased in T-Ca^2+^ and SK current densities in TRN neurons of adult *Gclm*-KO mice

Because of altered bursting profile and decreased T-Ca^2+^ current density at RMP in TRN neurons of young adult KO mice, we further investigated T-Ca^2+^ currents by measuring the amplitude of these currents when activated from different membrane potentials (−30 mV to −110 mV; ∆ = −5 mV). We found an overall decrease in T-Ca^2+^ current density in KO as compared to WT mice (Fig. 1G). As repetitive bursting of TRN neurons requires a coordinated activity between T-Ca^2+^ and SK channels, we also examined isolated SK currents. Similar to T-Ca^2+^, the SK current density was overall significantly lower in TRN neurons of KO compared to WT mice (Fig. 1H). However, the correlation between SK and T-Ca^2+^ currents was unaltered in KO mice (Fig. 1I). Altogether, these suggest that a decrease in T-Ca^2+^ currents accounts for the altered bursting profile of TRN neurons in adult *Gclm*-KO mice.

### Reduced expression of CaV3.3 channels in TRN of adult *Gclm*-KO mice

Given the decreased T-Ca^2+^ current density in TRN of adult *Gclm*-KO mice, we explored if this was linked to a reduced expression of T-Ca^2+^ channels, namely CaV3.2 and CaV3.3 [34]. We performed immunohistochemistry for CaV3.2 and CaV3.3 to quantify the expression of these two channels in the TRN (Fig. 1J–O). We found no genotype difference in number of CaV3.2-IR voxels, irrespective of the TRN sub-regions (Fig. 1K, L). By contrast, TRN of KO mice displayed significantly lower number of CaV3.3-IR voxels as compared to WT mice (Fig. 1N). The decreased CaV3.3 expression was most prominent in anterior and medial TRN sub-regions (Fig. 1O). These results suggest that reduced CaV3.3 expression contributes to the reduced T-Ca^2+^ current density in TRN neurons of adult *Gclm*-KO mice.

### Normal T-Ca^2+^ and SK currents in peripubertal *Gclm*-KO mice

**Fig. 2:**
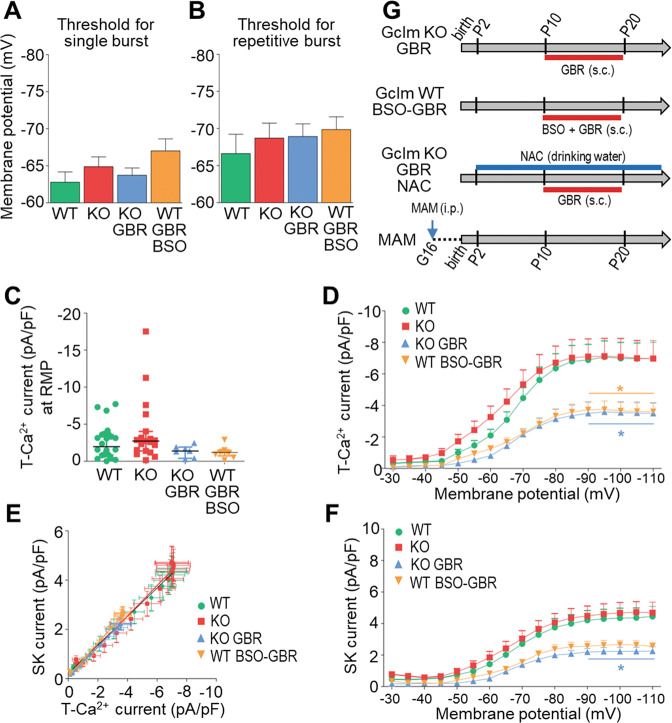
An additional oxidative challenge during early-life is required to precipitate hypofunction of T-Ca^2+^ channels in TRN neurons of young (PND20-30) mice. Threshold for the initial membrane potential required to induce single (**A**), or repetitive bursting (**B**), upon depolarization. Note a general trend for a more hyperpolarized membrane potential required to elicit burst firing in TRN of KO, KO GBR, WT BSO-GBR mice as compared to WT (WT *n* = 11; KO *n* = 12; KO GBR *n* = 10; WT BSO-GBR *n* = 11). **C** Density of T-Ca^2+^ currents activated at resting membrane potential (RMP), with a similar trend for lower current density in KO GBR and WT BSO-GBR as compared to WT mice (WT *n* = 24; KO *n* = 19; KO GBR *n* = 9; WT BSO-GBR *n* = 9) (Kruskal-Wallis, *p* = 0.062). **D** Density of T-Ca^2+^ currents activated from each of the initial membrane potentials, with significant overall difference between groups (*F* = 36.98 DFn = 3 DFd = 1029; *p* < 0.0001). Compared to WT mice, TRN neurons in KO GBR and WT BSO-GBR mice have smaller T-Ca^2+^ currents, particularly at the most hyperpolarized membrane potentials. **E** Correlation between T-Ca^2+^ and SK current densities (Spearman *r* = −0.98 for WT; −0.91 for KO; −0.97 for KO-GBR; −0.92 for WT BSO-GBR; p < 0.0001 for all). **F** Density of SK currents activated from each of the initial membrane potentials, with significant overall difference between groups (*F* = 37.84 DFn = 3 DFd = 961; *p* < 0.0001). Two-way ANOVAs with Bonferroni post-tests; *p < 0.05 **p < 0.01 ***p < 0.001. Data are presented as means ± s.e.m. **G** Timeline for the treatments received by the different animal groups of young mice: GBR-treated *Gclm*-KO (KO GBR), BSO + GBR treated WT (WT BSO-GBR) mice, NAC treatment of KO GBR mice (results in Fig. 3), and MAM mice (results in Fig. 4). Administration of the dopamine uptake inhibitor (GBR12909, GBR) from PND10 to PND20 was used as additional oxidative challenge. Administration of buthionine sulfoximine (BSO) from PND10 to PND20 was used to induce a transient GSH deficit in WT mice. MAM (methylazoxymethanol acetate) was injected to the mother at gestational day G16.

As we previously observed OxS and reduced PV expression in TRN of PND20 *Gclm*-KO [18], we examined T-Ca^2+^ currents in peripubertal (PND20-30) mice. The membrane potentials required to switch from tonic to single burst (Fig. 2A), or to repetitive bursting (Fig. 2B) were not statistically different between KO and WT mice. Moreover, T-Ca^2+^ current density at RMP (Fig. 2C) and at other membrane potentials (Fig. 2D) did not differ between genotypes. Likewise, SK current density was overall not different in KO and WT mice, and the correlation between T-Ca^2+^ and SK currents was maintained in these young KO mice (Fig. 2E, F). Thus, hypofunction of T-Ca^2+^ channels in TRN of *Gclm*-KO mice emerges later during late adolescence/early adulthood.

### Early-life oxidative challenge triggers T-Ca^2+^ channel hypofunction in peripubertal *Gclm*-KO mice

Next, we assessed the impact of an early-life oxidative challenge in mice with a compromised GSH system. The oxidative insult consisted of administration (from PND10 to 20) of the dopamine reuptake inhibitor GBR12909 (GBR) (Fig. 2G), which elevates extracellular dopamine and subsequently generates reactive oxygen species (ROS) and reactive compounds [35], mimicking to some extent dopamine release induced by environmental stress [36]. Such treatment exacerbates OxS in mice with compromised antioxidant systems [37]. GBR treatment did not affect significantly the threshold for single and repetitive bursting in peripubertal *Gclm*-KO mice when compared to age-matched WT mice, although a more hyperpolarized membrane potential tended to be required for eliciting repetitive bursting (Fig. 2A, B). However, T-Ca^2+^ current densities were significantly lower in GBR-treated KO as compared to WT mice, particularly at most hyperpolarized membrane potentials (Fig. 2C, D). Similar hypofunction of T-Ca^2+^ channels was found in peripubertal WT mice who had a transient BSO-induced GSH deficit combined with a GBR treatment (Fig. 2C, D, G). Both GBR-treated KO and GBR + BSO-treated WT mice displayed a concomitant decrease of SK current densities as compared to control mice (Fig. 2F). The correlation between T-Ca^2+^and SK currents was maintained in all groups (Fig. 2E). Together, this indicates that an early-life oxidative challenge under condition of GSH deficit precipitates T-Ca^2+^ channel hypofunction in peripubertal mice.

### N-acetylcysteine (NAC) prevents OxS and T-Ca^2+^ hypofunction

**Fig. 3:**
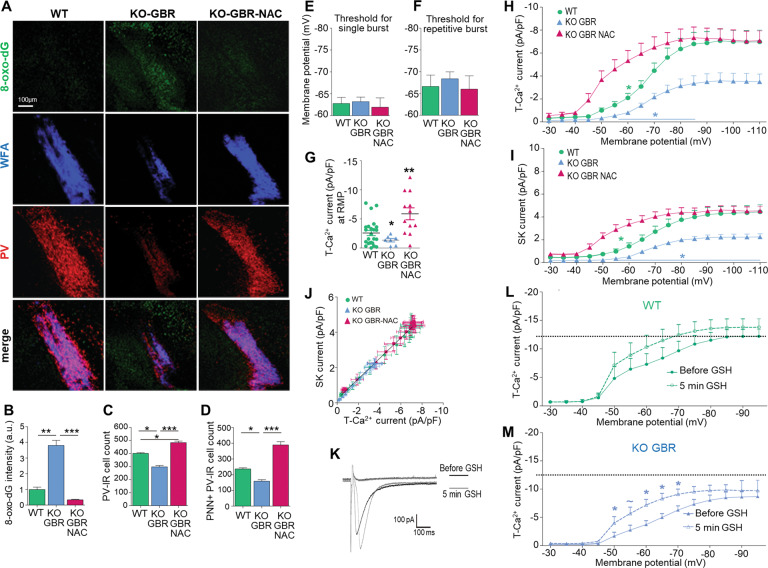
N-acetyl-L-cysteine (NAC) treatment prevents oxidative stress and hypofunction of T-Ca^2+^ channels in TRN neurons of young GBR-treated *Gclm*-KO mice. **A** Micrographs showing immunofluorescent labeling for 8-oxo-dG (oxidative stress marker, green), parvalbumin (PV, red), and WFA/PNN (blue) in the TRN of PND40 WT, KO GBR, and KO GBR-NAC mice. WFA/PNN: perineuronal net labeled with Wisteria floribunda agglutinin (WFA). NAC treatment in KO GBR mice decreases 8-oxo-dG labeling (**B**), increases the number of PV-IR neurons (**C**) and PNN + PV-IR neurons (**D**). Note that the numbers of PV-IR and PNN + PV-IR neurons are even higher in KO GBR-NAC as compared to WT mice. Data are from 5 mice per group. Threshold for the initial membrane potential required to induce single (**E**), or repetitive bursting (**F**) upon depolarization, with no significant differences between groups (WT *n* = 11; KO GBR *n* = 10; KO GBR-NAC *n* = 6). **G** Density of T-Ca^2+^ currents activated at resting membrane potential (RMP), with higher current density in KO GBR-NAC as compared to WT and KO GBR mice (*p* = 0.026 and *p* = 0.012, respectively, Kruskal–Wallis test followed by paired-wise comparisons and Bonferroni corrections) (WT *n* = 24; KO GBR *n* = 7; KO GBR-NAC *n* = 12). Density of T-Ca^2+^ (**H**) and SK (**I**) currents activated from each of the initial membrane potentials, with significant overall difference between groups for T-Ca^2+^ (*F* = 60.96 DF*n* = 2 DFd = 731; *p* < 0.0001) and SK currents (*F* = 75.16 DF*n* = 2 DFd = 697; *p* < 0.0001). The maximal currents activated from membrane potentials ≤ −80 mV are recovered by NAC treatment, with similar current density in TRN neurons of KO GBR-NAC and WT mice. Note however that T-Ca^2+^ and subsequently SK channels are more activated from depolarized membrane potentials (≥−65 mV) in TRN neurons of KO GBR-NAC as compared to both WT and KO GBR mice (shift in steady-state inactivation of T-Ca^2+^ channels). **J** Correlation between T-Ca^2+^ and SK current densities (Spearman *r* = −0.98 for WT; −0.97 for KO GBR; −0.91 for KO GBR-NAC; *p* < 0.0001). **K**–**M** Superfusion of 100 µM GSH in aCSF rapidly increases T-Ca^2+^ currents upon depolarization, particularly when the initial membrane potential of TRN neurons is ≤ −70 mV (shift in steady-state inactivation of T-Ca^2+^ channels). This effect of GSH is more pronounced in KO GBR (**M**) than in WT mice (**L**), suggesting more oxidized extracellular redox-sensitive sites of CaV3.2 channels in KO GBR as compared to WT mice. Horizontal dotted lines on (**L**) and (**M**) indicate maximal average current density for WT mice. Representative recordings of maximal T-Ca^2+^ currents before and after 5 min GSH application in aCSF are given in (**K**). aCSF: artificial cerebrospinal fluid. Two-way ANOVAs with Bonferroni post-tests; *p < 0.05 **p < 0.01 ***p < 0.001. Data are presented as means ± s.e.m.

We then examined whether a NAC treatment (Fig. 2G) could normalize T-Ca^2+^ currents in TRN neurons of GBR-treated KO mice. NAC lowered levels of the OxS marker, 8-oxo-dG, in TRN of peripubertal GBR-treated KO mice to levels found in age-matched WT mice (Fig. 3A, B). Concomitantly, NAC increased the number of PV-immunoreactive neurons and boosted WFA-labeled PNN (Fig. 3A, C, D). The number of PV-immunoreactive neurons following NAC treatment was even significantly higher than in WT mice and a similar trend was found for PNN. Such overshoot effect was also observed in NAC-treated WT mice (236 ± 7 and 411 ± 15 PV-immunoreactive neurons enwrapped with PNN in respectively WT (*n* = 5) and NAC-treated WT mice (*n* = 4); *t*-test, *p* < 0.001). Regarding the physiological properties, NAC did not alter the switch from tonic to burst firing (Fig. 3E, F), but strongly enhanced T-Ca^2+^ currents at RMP (Fig. 3G) in GBR-treated KO mice. Thus, NAC produced an overshoot effect on both T-Ca^2+^ currents and PV and PNN immunoreactivity. The quantification of T-Ca^2+^ currents activated from different membrane potentials (Fig. 3H) revealed that NAC fully normalized the maximal T-Ca^2+^ current density (currents activated at most negative membrane potentials) in TRN neurons of GBR-treated KO mice. However, we also observed a shift of the steady-state inactivation of T-Ca^2+^ currents to more depolarized membrane potentials in NAC-treated mice as compared to the other groups (Fig. 3H). This explains the strong activation of T-Ca^2+^ currents at RMP in NAC + GBR-treated KO mice (Fig. 3G). The effect of NAC on SK currents (Fig. 3I) paralleled that on T-Ca^2+^ currents (Fig. 3H). Thus, NAC did not modify the relationship between T-Ca^2+^ and SK currents (Fig. 3J). The NAC-induced shift of steady-state inactivation of T-Ca^2+^ currents suggested altered functional channel properties via an effect on extracellular redox-sensitive sites present in CaV3.2, but not CaV3.3. These sites can be reduced by redox agents such as GSH, enhancing the channel function [38]. We therefore tested the impact of a 5-min superfusion of GSH (100 µM) in aCSF on T-Ca^2+^ currents in WT and GBR-treated KO mice. GSH, which is not taken up by neurons [39], rapidly shifted the steady-state inactivation of T-Ca^2+^ currents in TRN neurons of both groups (Fig. 3K–M), similarly to NAC treatment (Fig. 3H). However, the GSH effect was more prominent in GBR-treated KO mice. In GBR-treated KO (Fig. 3M) but not WT mice (Fig. 3L), T-Ca^2+^ currents (at initial membrane potentials ≥ −70 mV) were significantly higher during GSH application than before. Collectively, this suggests more oxidized extracellular redox-sensitive sites on CaV3.2 in TRN neurons of GBR-treated KO as compared to WT mice and NAC treatment strongly protects these sites from oxidation.

### OxS reduced T-Ca^2+^ and SK current densities in TRN neurons of MAM mice

**Fig. 4:**
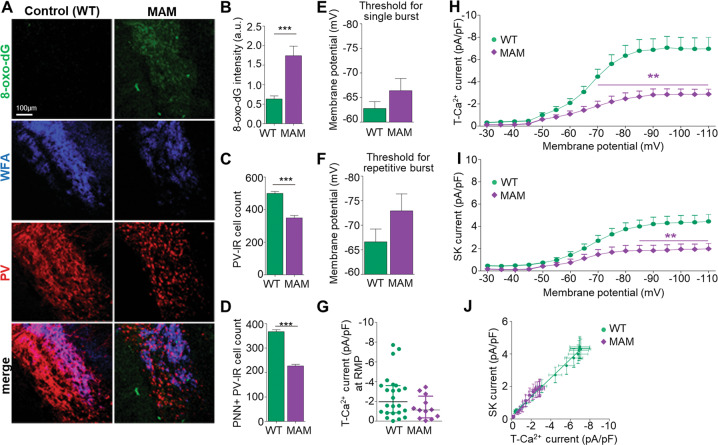
Concomitant presence of oxidative stress and hypofunction of T-Ca^2+^ channels in TRN of a neurodevelopmental mouse model relevant to psychosis (MAM mice). MAM mice are offspring from mothers who received a single injection of methylazoxymethanol acetate at gestational day 16. **A** Micrographs showing immunofluorescent labeling for 8-oxo-dG (oxidative stress marker, green), parvalbumin (PV, red), and WFA/PNN (blue) in the TRN of PND40 control and MAM mice. WFA/PNN: perineuronal net labeled with Wisteria floribunda agglutinin (WFA). Increased 8-oxo-dG labeling (**B**), decreased number of PV-IR neurons (**C**), and PNN + PV-IR neurons (**D**) in TRN of MAM compared to control mice. Data are from 4 mice per group. Threshold for the initial membrane potential of TRN neurons required to induce single (**E**), or repetitive bursting (**F**), upon depolarization. Note a trend for a more hyperpolarized membrane potential in order to induce bursting activity in TRN neurons of young (PND21-30) MAM (*n* = 13) as compared to control WT (*n* = 11) mice. **G** Density of T-Ca^2+^ currents activated at resting membrane potential (RMP) not significantly different in TRN neurons of MAM (*n* = 12) and control WT mice (*n* = 24). Density of T-Ca^2+^ (**H**) and SK (**I**) currents activated from each of the initial membrane potentials. Compared to control mice, TRN neurons in MAM mice exhibit overall significantly smaller T-Ca^2+^ (*F* = 73.63 DFn = 1 DFd = 578; *p* < 0.0001) and SK current densities (*F* = 61.58 DFn = 1 DFd = 544; *p* < 0.0001). **J** Correlation between T-Ca^2+^ and SK current densities (Spearman *r* = −0.96 for WT and −0.99 for MAM; *p* < 0.0001 for both groups). Unpaired *t*-tests and two-way ANOVAs with Bonferroni post-tests; *p < 0.05 **p < 0.01 ***p < 0.001. Data are presented as means ± s.e.m.

We then examined whether T-Ca^2+^ channels in TRN neurons were also affected in another rodent model relevant to SZ, MAM mice (see Fig. 2G). Originally studied in rats [40], a late prenatal methylazoxymethanol acetate (MAM) administration causes disruption of normal brain maturation leading to histological, neurophysiological, and behavioral deficits analogous to SZ [41–43], including PV cell network anomalies [37, 42]. As for GBR-treated *Gclm*-KO mice, the TRN of peripubertal (PND20-30) MAM mice showed significant increase in 8-oxo-dG, decreased number of PV-IR neurons, and weakened WFA-labeled PNN (Fig. 4A–D). Regarding the spiking properties of TRN neurons, the membrane potential required to trigger bursting in MAM mice was slightly more negative compared to control mice (Fig. 4E, F). T-Ca^2+^ current density at RMP tended to be weaker in MAM mice (Fig. 4G). Remarkably, we found a significant overall decrease of T-Ca^2+^ current density in MAM mice, especially at most hyperpolarized membrane potentials (≤ −70 mV) (Fig. 4H). SK current density was concomitantly reduced, particularly for membrane potentials ≤ −85 mV (Fig. 4I). The correlation between T-Ca^2+^ and SK currents was however maintained in MAM as compared to control mice (Fig. 4J). The finding that OxS is accompanied with a decrease of T-Ca^2+^ currents in TRN neurons of MAM mice, similarly to GBR-treated *Gclm*-KO mice, further supports that OxS causes hypofunction of T-Ca^2+^ channels.

## Discussion

We have found a novel mechanistic link between OxS and hypofunction of T-Ca^2+^ channels in TRN of two mouse models pertinent to SZ: *Gclm*-KO and MAM mice. In *Gclm*-KO mice who display OxS in the TRN throughout their postnatal life [24], we observed a progressive decrease of T-Ca^2+^ currents in TRN neurons at early adulthood together with a reduction of CaV3.3 expression. However, an oxidative challenge mimicking an environmental insult during early-life causes a decrease of T-Ca^2+^ currents in TRN neurons of peripubertal *Gclm*-KO mice, which is prevented by NAC. Likewise, TRN neurons of peripubertal MAM mice show similar concomitant presence of OxS and reduced T-Ca^2+^ currents. Together, these suggest that OxS-mediated T-Ca^2+^ channel hypofunction in TRN neurons could represent a common and convergent mechanism contributing to early thalamo-cortical anomalies relevant to the pathogenesis of SZ.

TRN neurons display high neuronal activity (up to ~40 action potentials/s) in freely-behaving mice [28]. This requires a strong energy metabolism coupled with efficient antioxidant and neuroprotective mechanisms. As for prefrontal fast-spiking PV interneurons [37], a perturbation of this homeostatic coupling, which could arise from various genetic and environmental aetiologies, causes OxS and subsequently structural and functional alterations of the TRN. TRN neurons are indeed prone and vulnerable to OxS in both *Gclm*-KO and MAM mice, but also in PV-KO mice [44], and upon NMDAR blockade [26]. This vulnerability occurs already at young age, affecting TRN PV-IR neurons. In *Gclm*-KO mice, decrease of PV-expressing TRN neurons precedes PV deficit in other brain regions [45]. Likewise in the MAM model, decreased PV expression in TRN is already observed at peripuberty, but only emerges at early adulthood in prefrontal cortex [46].

It is tempting to speculate that TRN neurons would reduce their excitability and bursting activity under OxS conditions via a negative regulation of T-Ca^2+^ channels, but at the expense of a reduced inhibitory control and modulation of thalamo-cortical circuits. In young adult *Gclm*-KO as compared to WT mice, TRN neurons show weaker T-Ca^2+^ currents resulting in lower probability to generate bursts of action potentials at RMP and smaller SK currents. The concomitant decrease of T-Ca^2+^ and SK currents is responsible for the more hyperpolarized membrane potential required to induce repetitive bursting in KO mice. This corroborates previous observations that TRN neurons are less inclined to elicit burst firing at physiologically relevant conditions in adult *Gclm*-KO as compared to WT mice [24].

In peripubertal *Gclm*-KO mice however, both T-Ca^2+^ and SK currents are not affected despite the presence of OxS and reduced PV expression [24]. At this young age, the OxS levels may be insufficient to impact T-Ca^2+^ channels and/or compensatory mechanisms resulting from the constitutive GSH deficit [24] may still efficiently stem OxS-mediated hypofunction of these channels. Maintaining robust activity of T-Ca^2+^ channels in TRN neurons of young individuals may be crucial for proper maturation of thalamo-cortical circuits. However, an early-life oxidative challenge by the dopamine reuptake inhibitor GBR precipitates hypofunction of T-Ca^2+^ channels in TRN neurons of peripubertal *Gclm*-KO mice, but also WT mice with a transient GSH deficit imposed by a treatment with the GSH synthesis inhibitor, BSO. Nevertheless, as opposed to adult mice, the membrane threshold required for burst firing was not significantly different between the various groups of young mice. This may be due to the higher T-Ca^2+^ current density in TRN neurons of young as compared to adult mice. Thus, these currents may be sufficiently powerful to elicit burst firing from membrane potentials close to RMP, even in mice displaying significantly weaker T-Ca^2+^ currents as compared to young WT mice. In NAC + GBR-treated KO mice, the membrane threshold required for burst firing was also not significantly different from NAC-untreated mice despite the enhanced T-Ca^2+^ currents activated from a membrane potential more positive than RMP. The reasons are unclear but could involve afferent regulatory circuits.

The causal role of OxS in the mechanism underlying T-Ca^2+^ channel hypofunction emanates from the facts that the antioxidant NAC normalizes the maximal amplitude of T-Ca^2+^, abolishes OxS, boosts PV immunoreactivity, and strengthens PNN deficits in TRN of GBR-treated *Gclm*-KO mice. Of note, these effects were due to NAC own antioxidant properties, as similar NAC treatment failed to increase brain GSH levels in *Gclm*-KO and WT mice [47]. Although we did not assess the effect of NAC on T-Ca^2+^ currents in MAM mice, a recent study shows that NAC treatment in MAM rats also reduced OxS, restored PV expression and PNN integrity, and most importantly recovered proper TRN-mediated feedforward inhibition likely via the reinstatement of high-frequency bursting due to the normalization of PNN [27]. This study highlighted the role of TRN to stabilize the firing pattern within the infralimbic cortex-TRN-reuniens nucleus circuit [48] which modulates VTA dopamine neuron activity. Thus, these data support that NAC corrects for abnormal TRN activity and excitability, also likely via an action on T-Ca^2+^ channels in MAM rodent models.

The mechanism underlying OxS-mediated hypofunction of T-Ca^2+^ channels in *Gclm*-KO and MAM mice warrants however further investigation. In all investigated animal models, the larger effect is observed when T-Ca^2+^ channels are activated from the most hyperpolarized membrane potentials, namely when all channels are presumably de-inactivated. Thus, the weaker maximal T-Ca^2+^ current density in *Gclm*-KO and MAM models as compared to control mice may reflect a lower expression of CaV3.3 channels, as observed in TRN of young adult *Gclm*-KO mice. However, we cannot rule out other mechanisms, including negative redox modulation of CaV.3.2 activity. Redox agents regulate the function of CaV3.2, but not Cav3.3, by acting on the extracellular loops of domain I via metal-catalized oxidation requiring a histidine and redox modulation of cysteines. This modifies the kinetics of activation/inactivation of the channels [49, 50]. Thus, oxidizing and reducing molecules respectively decrease and enhance CaV3.2-dependent currents in TRN neurons [51]. We found that extracellular GSH superfusion causes rapid and significant enhancement of T-Ca^2+^ currents at depolarized membrane potentials (≥−70 mV) in GBR-treated KO, but not WT mice. This points that CaV3.2 extracellular redox sites are more oxidized in TRN neurons of GBR-treated KO as compared to WT mice. NAC treatment causes a shift of the steady-state inactivation of T-Ca^2+^ currents in GBR-treated KO mice, similarly to the effect of an extracellular GSH superfusion. This indicates that NAC protects the redox-sensitive sites from oxidation, keeping them in a highly reduced state even during the slicing procedure and throughout the ex-vivo experiments. At relatively depolarized membrane potentials, T-Ca^2+^ current amplitudes are larger in NAC + GBR-treated KO mice as compared to GBR-treated KO but also WT mice. This parallels the boosting of PNN by NAC, even in WT mice. Thus, the NAC-induced protection of the redox-sensitive sites from oxidation may arise from the NAC-induced robust strengthening of the PNN, which constitutes a protective shield against OxS [52]. Interestingly, enzymatic removal of the PNN in anterior TRN disrupts its inhibitory action on the nucleus reuniens, consequently resulting in increased number of active VTA dopaminergic neurons [27].

The reduced excitability and burst firing of TRN neurons, resulting from OxS-mediated decrease of T-Ca^2+^ currents, could affect thalamo-cortical activity in-vivo and affect circadian rhythm regulation. Corroborating our ex-vivo findings, reduced neuronal activity and burst density of TRN neurons is also observed during NREM sleep in freely-behaving *Gclm*-KO mice who show fragmented sleep with a prominent impact on NREM sleep, altered thalamo-cortical network dynamics, and a lack of homeostatic upregulation of spindle rate and delta oscillations during recovery from sleep deprivation [28]. Likewise, MAM rats display fragmented sleep, reduced length of NREM bouts with a small decrease in spindle density [53].

To summarize, OxS-mediated T-Ca^2+^ channel hypofunction in TRN, via reduced CaV3.3 expression and oxidation of redox-sensitive sites on CaV3.2, could represent a convergent mechanism contributing to early thalamo-cortical anomalies [54, 55] and SZ pathogenesis. Sleep disturbance constitutes one hallmark of prodromal features preceding a first psychotic episode [56, 57]. Likewise, sleep spindle deficits are reported in non-psychotic first-degree relatives and during early course of the disorder [58–60]. Together with genetic associations of CACNA1I (encoding CaV3.3) and to a lesser extent CACNA1H (encoding CaV3.2) with SZ [33, 61–63], this suggests that T-Ca^2+^ channel dysfunction in TRN could represent an early pathological feature of this psychiatric disorder. The reversal effect of NAC paves the way for novel early intervention strategies.

## Supplementary information

Supplemental information
