# Supplementary material for: Developmental oxidative stress leads to T-type Ca2+ channel hypofunction in thalamic reticular nucleus of mouse models pertinent to schizophrenia
Source: Mol Psychiatry. 2022 Jan 25;27(4):2042–51. doi: 10.1038/s41380-021-01425-2 (PMC9126813; doi:10.1038/s41380-021-01425-2)
Supplement: Supplementary file 1 — Supplemental information [file 41380_2021_1425_MOESM1_ESM.docx]

**SUPPLEMENTAL INFORMATION**

## METHODS

**Animal models**

Mice were housed under a 12-hour light-dark cycle in groups of 3–5 individuals/cage, and had access to food and water ad libitum. Experiments were approved by the Local Veterinary Office (license VD3285). In this study, we used different animal models to assess the effect of developmental oxidative stress:

*Gclm-KO mice:* These mice ^1^ were initially provided by T. Dalton (University of Cincinnati) and backcrossed with C57BL/6J mice over >10 generations. All breeding mice were obtained from heterozygotes. Experimental *Gclm*-KO mice were offsprings from *Gclm*-KO males and heterozygote females, while *Gclm* +/+ (WT) were obtained from breeding WT males and females.

*GBR-treated Gclm-KO mice and GBR+BSO-treated WT mice* (Fig. 2A): To assess the effect of an early-life additional oxidative insult in mice with a redox dysregulation, we administrated the dopamine reuptake inhibitor GBR-12909 (GBR, Bio Trend, Germany) in either preweaned *Gclm*-KO mice or WT mice with a transient GSH deficit induced by the specific inhibitor of GSH synthesis, buthionine sulfoximine (BSO, Sigma-Aldrich, Switzerland). GBR-12909 treatment results in excess extracellular dopamine levels generating ROS and OxS in animals with a weakened antioxidant system ^2^. From postnatal days (PND)10 to PND20, GBR-12909 (5 mg/kg, i.p.; in 0.1M phosphate buffer saline, pH 7.3) was injected daily in *Gclm*-KO mice and every other day in WT mice that received a daily injection of BSO (3.8 mmol/kg, s.c.).

*MAM mice* (Fig. 2A): Female WT (C57BL/6JRj) mice were received from Janvier-Labs (France) at gestational day 9 and injected intraperitoneally on gestational day 16 with either 25 mg/kg methylazoxymethanol acetate (MAM, MRIGlobal Chemical Carcinogen, USA) or the vehicle solution (0.9% NaCl). The offsprings of MAM- or NaCl-treated females were used for experiments.

Note that for both electrophysiology and immunohistology experiments, the allocation of an animal to a specific treatment was done arbitrary but without using a method of randomization.

**N-Acetylcysteine (NAC) treatment**

NAC treatment was given to GBR-treated *Gclm*-KO mice (Fig. 2A). NAC (Fluimucil, Zambon, Switzerland) was provided in drinking water (2.4 g/l) to the lactating *Gclm* mothers from PND2 to weaning throughout the GBR treatment duration (from PND10 to 20). After weaning and until sacrifice, GBR-treated *Gclm*-KO mice continued to have access to NAC in drinking water. NAC solution was renewed every other day

**Electrophysiology experiments**

Both males and females were used at two age groups: peripubertal (from PND20 to PND30) or young adult (from PND60 to PND75).

**Slice preparation**

Mice aged from 20 to 30 days were deeply anesthetized with isoflurane, then decapitated. The brain was removed and placed into ice-cold (4°C) sucrose-based artificial cerebrospinal fluid (aCSF) containing (in mM): 205 sucrose, 2 KCl, 7.0 MgCl_2_, 26 NaHCO_3_, 1.2 NaH_2_PO_4_, 11 D-glucose, and 0.5 CaCl_2_ (pH 7.3) bubbled with 95 % O_2_ and 5 % CO_2_. The two hemispheres were separated and cut into serial horizontal slices (300-µm thick) using a vibratome (Campden 7000smz, Campden Intruments, UK). Slices were incubated at 37 °C in aCSF containing (in mM): 130 NaCl, 3 KCl, 2.5 CaCl_2_, 1.3 MgSO_4_, 0.6 NaH_2_PO_4_, 25 NaHCO_3_, 10 glucose (pH 7.3) bubbled with 95 % O_2_ and 5 % CO_2_, for a 30 min recovery period, then stocked in the same solution at room temperature.

For mice aged from 60 to 75 days, slices were cut in chilled N-Methyl-D-glucamine (NMDG) solution containing (in mM): 93 NMDG, 2 KCl, 7.0 MgSO_4_, 26 NaHCO_3_, 1.2 NaH_2_PO_4_, 11 D-glucose, 0.5 CaCl_2_, ascorbic acid, Na-pyruvate and L-cysteine, 4°C, pH = 7.3, bubbled with 95 % O_2_ and 5 % CO_2_. The slicing plan was coronal to facilitate the visibility of cells due to the high density of fibers in the TRN. The slices were incubated in the NMDG solution at 37°C for 20 min to allow recovery, then stored in oxygenated aCSF at room temperature. Slices were transferred into the recording chamber (volume ≈ 1 ml) and held down with a C-shape platinum wire supporting transverse nylon fibers. The chamber was mounted on an upright microscope (Olympus BX51WI, Switzerland) and linked to a IR camera (PCO, Germany). Slices were continuously perfused at 3.0 ml/min with oxygenated aCSF maintained at room temperature (≈ 25°C). Neurons were recorded under visual control using X63 water-immersion objective lens added with a x2 magnifier (Luigs & Neumann, Germany), under a combined infrared and differential interference.

Borosilicate glass tubes with filament (1.5mm 0.86mm 7.5cm, Sutter Instrument, Novato, CA, USA) were pulled with a DMZ Zeitz puller (Germany) to obtain patch pipettes of 4-7 MΩ when filled with an intracellular solution containing (in mM): 140 K-gluconate, 0.1 EGTA, 10 Hepes, 10 KCl, 10 Phosphocreatinin, 4 Mg-ATP, 0.4 Na-GTP_,_ neurobiotin (0.05 %, Vector Laboratories, Burlingame, CA, USA), 300 mOsm, pH 7.3). For Glutathione (GSH) experiments, K-gluconate was replaced with Cs-gluconate to block SK channels, and avoid interactions between the applied drugs. The solution contained 127 Cs-gluconate, 10 HEPES, 2 BAPTA, 6 MgCl2, 10 phosphocreatinin, 2 Mg-ATP, 0.4 Na-GTP.

**Patch-clamp recordings**

Acquisitions were performed using Clampex 10.4 software (Molecular Devices, Sunnyvale, CA, USA) connected to a Multiclamp 700B amplifier (Molecular Devices, Sunnyvale, CA, USA) via a Digidata 1550 digitizer (Molecular Devices, Sunnyvale, CA, USA). Whole-cell recordings were performed in both voltage clamp (VC) and current clamp (CC) modes. VC data were low pass filtered at 3 kHz and digitized at 10 kHz. Series resistance was monitored throughout the experiments and was not compensated. Data were discarded if series resistance varied of more than 20% from its initial value, or if the neurons had a resting membrane potential more depolarized than -50 mV. The resting membrane potential (RMP) and the firing pattern were studied in the CC mode. The firing profile at RMP was determined by a series of pulses of incremental amplitude (-40 to + 40 pA, ∆=10 pA, 1 s). A burst was defined as a high frequency phasic spiking (interspike frequency >100 Hz) followed by an afterhyperpolarization (AHP) period. To further determine the precise membrane potential at which the neuron switches from tonic firing to single bursting and then to repetitive bursting, the following CC protocol was used: the current was clamped to maintain the membrane potential at -50 mV, and a series of small hyperpolarizing current pulse steps (-5 to -60 pA, 2 s) followed by a depolarizing step (20 pA, 1 s) were applied. The voltage protocols used to isolate the T-Ca^2+^currents and SK currents were previously described ^3^. Briefly in presence of tetrodotoxin (TTX; 1 µM; Sigma-Aldrich, Switzerland), the membrane potential was clamped at -60 mV, and 1 s voltage steps (from -30 mV to -110 mV) followed by a short depolarization step from -110 to -40 mV were applied. This same protocol was then repeated in presence of TTX and 100 nM apamin, a selective SK channel blocker, in order to isolate T-Ca^2+^ currents. Isolated SK-currents were obtained from the subtraction of the recorded currents under TTX+apamin from the currents recorded under TTX. All data were analyzed offline using Clampfit 10.0 software (Axon Instrument Molecular Devices, Sunnyvale, CA, USA). The liquid junction potential, estimated at -5 mV based on the liquid junction potential calculator in Clampex (Axon Instruments, Molecular Devices) was not corrected.

To test the redox effects on the T-Ca^2+^ currents, we recorded T-Ca^2+^ currents just before and 5 min following application of 100 µM of L-glutathione (GSH, Sigma-Aldrich, Switzerland) freshly diluted in aCSF. For these recordings, the intracellular solution contained Cs-gluconate (see previous chapter for detailed composition of the solution). In our study, most recorded TRN neurons were presumably PV neurons, as their burst firing property and T-Ca^2+^ current density resembles more those of PV than somatostatin TRN neurons ^4^.

While the experimenter was aware of the mice genotype and/or treatment during recordings, analyses of the recorded cells were performed without knowledge of the genotype and/or treatment.

**Immunohistochemistry (IHC)**

**PV / WFA / 8-oxo-dG immunohistology**

For the immunohistochemistry (IHC) experiments, we only used male animals. Mice were deeply anesthetized with 5mg/kg pentobarbital, and perfused transcardially with 4% paraformaldehyde in PBS, pH 7.3. The brains were extracted, further post-fixed for 2 hours, and transferred to a 30% sucrose 0,5% Na-azide PBS solution. Coronal frozen sections (30 µm) were cut with a microtome (Microm HM440E, Thermo Scientific instrument, Germany), and stored in 30% ethylene glycol at -20 °C until use. PV / WFA / 8-oxo-dG triple immunofluorescence protocol was done as previously described 5. Briefly, brain sections (2-3 per mouse) were blocked and permeabilized, then incubated for 48h in primary antibodies solution with mouse monoclonal anti-8-oxo-dG (1:400; # 4354-MC-050, AMS Biotechnology, Switzerland); rabbit polyclonal anti-parvalbumin (1:2500; PV27, Swant, Switzerland) and biotin-conjugated lectin Wisteria floribunda Agglutinin (WFA,1:2000; L1516, Sigma-Aldrich, Switzerland). Corresponding secondary antibody conjugates were applied (goat anti-mouse (1:300; AF488; #A-11001, Life Technologies, USA), goat anti-rabbit IgG (1:300; CY3; #AP132C, Chemicon International, USA) and streptavidin 405 conjugate (1:300; AF405; # S-32351, Life Technologies, USA), to visualize 8-oxo-dG, PV, and WFA labeling, respectively.

**CaV3.2 / PV and CaV3.3 / PV immunohistology**

CaV3.2 and CaV3.3 expression was assessed by immunohistology in anterior, medial and posterior TRN (3 sections per region per animal, 4-5 animals per group). The protocol was adapted from previously described method ^6^ for antigen retrieval. Briefly, a 5-min bath in 1% sodium dodecyl sulfate (SDS) was applied. For CaV3.2 labelling, all IHC steps were first performed without a membrane permeabilization step, as the target of the primary antibody (rabbit anti-CaV3.2, # SC-25691, 1:100, Santa Cruz Biotechnology, USA) was located in an extracellular domain of the channel. Then, slices were permeabilized with 0.5 % Tween to complete the IHC for PV (sheep anti-PV, 1/3000, #AF5058, R&D systems, Canada). For CaV3.3 labelling, the IHC steps were performed at the same time for both rabbit anti-CaV3.3 (1:100, #ACC-009, Alomone labs, Israel) and sheep anti-PV (1:3000, R&D systems) following an initial blocking and permeabilization step in 0.1M PBS, 0.5% Tween, 2% NGS). Corresponding secondary antibody conjugates were applied: goat anti-rabbit AF488 and donkey anti-sheep AF594 (1:300, respectively #ab150077 and ab150180, Abcam, UK) to visualize, respectively, CaV (CaV3.2 or CaV3.3) and PV labelling.

**Confocal imaging and image analysis**

Damaged tissue sections were excluded and only sections with fully intact TRN were analyzed per animal. All brain sections were visualized and processed with a Zeiss confocal microscope equipped with x10, x40 and x63 Plan-NEOFLUAR objectives. All peripherals were controlled with LSM 710 Quasar software (Carl Zeiss AG, Switzerland). Z stacks of 14 images (with a 1µm interval) were scanned (1024 × 1024 pixels) with ×63 oil immersion objective. Analysis was performed using IMARIS 9.2 software (Bitplane AG, Switzerland). Quantification of PV (intensity and number of PV-immunoreactive cells, WFA labelling (WFA labeling intensity and PV-IR cells surrounding with WFA), and 8-oxo-dG was performed in the TRN as previously5. Analysis of CaV was done as followed. Three sections containing respectively anterior, medial, and posterior TRN (at ~ Bregma: -0.82 to -0.94 mm, and Interaural: 2.98 to 2.68 mm) were used to do CaV3.3- and CaV3.2-immunoreactive (IR) puncta quantification. The region of interest (ROI) was defined and marked throughout the inner 12 images of Z-stacks to isolate regional sub-volumes of TRN (2.62x106 µm3), in which the number of CaV-IR voxels were quantified. Note that the immunohistological procedure of the tissue sections, the image acquisition and the analyses of the images were performed blindly without knowledge of the genotype and/or treatment.

**Statistical analysis**

For the electrophysiological experiments, no statistical method was used to estimate prior to the experiments an appropriate sample size for detecting a pre-specified effect size. For the immunohistological experiments and based on previous findings, the number of animals per group and sections per animal were chosen to detect ~ 25% change in number of PV-IR and PNN+PV-IR cells ,and ~ 75% change in 8-oxo-dG intensity with a power of 80% at a significant α-value set to p = 0.05. Regarding Cav3.3-IR and Cav3.2-IR, a similar number of animals per group was used without determining a pre-specified effect size.

Graphs and statistical analyses were performed using SPSS (IBM analytics, USA) and GraphPad Prism (v6.0, GraphPad Inc., La Jolla, CA, USA) softwares. Typically in the case of normal distributions of the data (checked with the Shapiro-Wilk Test), unpaired Student’s t-test (with Welch correction when unequal variances) was used to compare values between two groups. Comparisons of more than two groups were performed using one and two-way analysis of variance (ANOVA) followed by the Bonferroni Multiple Comparison Test according to the experimental design. Non parametric tests (Mann-Whitney and Kruskal-Wallis tests) were used when data did not fit normal distributions. When post-hoc analyses were needed following the Kruskal-Wallis test, the Dunn's multiple comparison test was used. Spearman's correlation was used to test the correlation between T-Ca2+ and SK current densities. P < 0.05 was considered statistically significant. Figures were made using CorelDraw Graphics software (v12.0, Ottawa, Canada) and Inkscape vector graphics editor.

**REFERENCES**

1 Yang Y, Dieter MZ, Chen Y, Shertzer HG, Nebert DW, Dalton TP. Initial characterization of the glutamate-cysteine ligase modifier subunit Gclm(-/-) knockout mouse. Novel model system for a severely compromised oxidative stress response. *JBiolChem* 2002; **277**: 49446-49452.

2 Steullet P, Cabungcal JH, Coyle J, Didriksen M, Gill K, Grace AA *et al.* Oxidative stress-driven parvalbumin interneuron impairment as a common mechanism in models of schizophrenia. *Mol Psychiatry* 2017; **22**: 936-943.

3 Huguenard JR, Prince DA. A novel T-type current underlies prolonged Ca(2+)-dependent burst firing in GABAergic neurons of rat thalamic reticular nucleus. *J Neurosci* 1992; **12**: 3804-3817.

4 Clemente-Perez A, Makinson SR, Higashikubo B, Brovarney S, Cho FS, Urry A *et al.* Distinct Thalamic Reticular Cell Types Differentially Modulate Normal and Pathological Cortical Rhythms. *Cell Rep* 2017; **19**: 2130-2142.

5 Steullet P, Cabungcal JH, Bukhari SA, Ardelt MI, Pantazopoulos H, Hamati F *et al.* The thalamic reticular nucleus in schizophrenia and bipolar disorder: role of parvalbumin-expressing neuron networks and oxidative stress. *Mol Psychiatry* 2018; **23**: 2057-2065.

6 Salameh S, Nouel D, Flores C, Hoops D. An optimized immunohistochemistry protocol for detecting the guidance cue Netrin-1 in neural tissue. *MethodsX* 2018; **5**: 1-7.
